# Territorial Socioeconomic Vulnerability and Clinical Outcomes in Pediatric Cancer at a Colombian Referral Center

**DOI:** 10.3390/children13081094

**Published:** 2026-08-18

**Authors:** Claudia Galeano-Páez, Ana Peñata-Taborda, Hugo Brango, Pamela Londoño-García, Javier Ospina-Martínez, Jaime Polo, Gabriela Jaramillo-Bedoya, Lyda Espitia-Pérez

**Affiliations:** 1 Grupo de Investigación Biomédica y Biología Molecular, Facultad de Ciencias de la Salud, Universidad del Sinú, Montería 230001, Córdoba, Colombia; investigador01gibm@unisinu.edu.co (A.P.-T.); pamelalondonog@gmail.com (P.L.-G.); gjaramillob01@gmail.com (G.J.-B.); lydaespitia@unisinu.edu.co (L.E.-P.); 2 Instituto de Estudios en Desarrollo, Economía y Sostenibilidad (IDEEAS), Universidad Tecnológica de Bolívar, Cartagena 130001, Bolívar, Colombia; 3 Departamento de Matemáticas, Facultad de Educación y Ciencias, Universidad de Sucre, Sincelejo 700003, Sucre, Colombia; hugo.brango@unisucre.edu.co; 4 Unidad de Estudios Clínicos e Investigación, Clínica IMAT Oncomédica AUNA, Montería 230001, Córdoba, Colombia; javier.ospina@imatoncomedica.com (J.O.-M.); jaimeolomartinez@gmail.com (J.P.)

**Keywords:** pediatric oncology, multidimensional poverty, social determinants of health, clinical outcomes, health disparities

## Abstract

**Highlights:**

**What is the main finding?**

- Childhood cancer cases in Córdoba and Sucre showed high territorial socioeconomic vulnerability, with marked rural–urban deprivation; older age, non-hematologic malignancies, and subsidized health insurance were associated with poorer outcomes, while multidimensional poverty was not independently associated after adjustment.

**What is the implication of the main finding?**

- Multidimensional poverty indicators and a One Health perspective may help identify structurally vulnerable territories and guide integrated strategies addressing clinical care, social determinants, environmental conditions, and health-system inequalities in pediatric oncology.

**Abstract:**

**Background/Objectives**: Childhood cancer outcomes are influenced by clinical, socioeconomic, and territorial factors, particularly in resource-limited settings. This study characterized pediatric cancer patients from Córdoba and Sucre, Colombia, and evaluated factors associated with clinical outcomes in a regional referral center. **Methods**: A retrospective hospital-based study included 434 patients aged 0–18 years diagnosed between 2018 and 2024. Sociodemographic, clinical, and territorial socioeconomic variables were analyzed using the Multidimensional Poverty Index (MPI). Clinical outcomes were classified as favorable, non-favorable, or death. Firth-penalized logistic regression models were used to assess adjusted associations. **Results**: Hematologic malignancies predominated (65.9%), with acute lymphoblastic leukemia as the most frequent diagnosis. Most patients came from municipalities with moderate or high multidimensional poverty (85.2%), and the rural territories of origin showed greater deprivation in education, employment, housing, water access, and sanitation. Favorable outcomes occurred in 82.9% of patients, while 8.5% had non-favorable outcomes and 8.5% died. Older age and non-hematologic malignancies were associated with non-favorable outcomes. Mortality was associated with older age, subsidized health insurance, and non-hematologic malignancies. MPI and rural residence were not independently associated with outcomes after adjustment. **Conclusions**: Although MPI was not independently associated with clinical outcomes, it identified substantial territorial deprivation. Integrating clinical and territorial indicators may support equity-oriented surveillance and pediatric oncology interventions.

## 1. Introduction

Childhood cancer is a major global public health priority because of its disease burden, mortality, and profound inequalities in clinical outcomes. According to the Global Burden of Disease 2023 study, approximately 377,000 new cases and 144,000 deaths were estimated among children and adolescents aged 0–19 years, corresponding to 11.7 million disability-adjusted life years (DALYs) [1]. Although global mortality decreased by 27.0% between 1990 and 2023, this progress has been uneven and concentrated mainly in countries with higher socioeconomic development [2]. This burden also reflects a critical dimension of health sustainability: childhood cancer accounts for a disproportionate loss of premature life in lower-development settings, where countries with a low Sociodemographic Index present the highest DALY rates [3]. The most critical gap is reflected in survival: while more than 80% of children with cancer can be cured in high-income countries, in many low- and middle-income countries this proportion remains close to or below 30% [4]. In these contexts, unfavorable childhood cancer outcomes reflect cumulative barriers related to diagnosis, access, treatment continuity, and quality of care [5,6,7,8].

In Latin America and the Caribbean, although progress has been documented in cancer control policies, regional cooperation, and early diagnosis strategies, substantial inequalities persist between countries and within national territories [9,10]. Pediatric patients with cancer and their families face logistical, economic, geographic, and administrative barriers that affect timely diagnosis, treatment initiation, and therapeutic continuity [10]. In addition, access to, availability of, and quality of essential pediatric oncology medicines remain heterogeneous across the region [11].

In Colombia, childhood cancer remains a challenge because of its epidemiological burden and territorial inequalities in access to specialized care. National studies have estimated a mean annual incidence of approximately 33 childhood leukemia cases per million children under 15 years of age and 44 non-leukemia tumors per million children, with important geographic variation [12,13]. Likewise, VIGICANCER reported, in a cohort of 9986 patients under 19 years of age, a 5-year overall survival of 63.3% and a 5-year event-free survival of 57.9%, values below those observed in high-income countries [14].

These inequalities are particularly relevant in rural and socially vulnerable territories, where distance to specialized centers, fragmentation of services, administrative barriers, and family economic constraints may shape the clinical trajectory of children with cancer. Evidence shows that delays in pediatric oncology care in low- and middle-income countries are determined by individual, family, sociocultural, geographic, and health-system factors [7]. In this regard, interventions focused on health worker education, early referral, and the use of digital tools have shown potential to reduce diagnostic delays [15,16]. The departments of Córdoba and Sucre, located in the Colombian Caribbean region, provide a relevant setting for studying these inequalities. Both territories are characterized by high rurality, persistent socioeconomic vulnerability, and dependence on regional referral centers for pediatric oncology diagnosis and treatment. In these contexts, isolated indicators such as insurance status, income, or area of residence are insufficient to capture the structural conditions affecting children, their families, and their communities.

The Multidimensional Poverty Index (MPI) offers a more comprehensive approach to territorial socioeconomic vulnerability. Based on the Alkire–Foster methodology, it identifies simultaneous deprivations in education, childhood and youth conditions, health, employment, housing, and access to public services [17,18]. Consistent with One Health approaches that recognize the interconnected influence of social, environmental, and health-system conditions on health outcomes [19] the MPI captures multiple contextual dimensions that may shape access to care, treatment continuity, and clinical outcomes in pediatric cancer. Unlike unidimensional indicators, the MPI recognizes that territories may experience different combinations of deprivation, which may influence access to care, treatment continuity, and clinical outcomes in pediatric cancer [18,20]. However, its use as a territorial variable in childhood cancer research remains limited, particularly in peripheral departments such as Córdoba and Sucre, where specific epidemiological evidence is scarce [12,14].

Therefore, the primary objective of this study was to characterize the clinical, territorial, and socioeconomic profile of children and adolescents with cancer from Córdoba and Sucre who were treated at a regional referral center in northern Colombia, using municipality-level Multidimensional Poverty Index data as a contextual measure of vulnerability. As an exploratory secondary objective, we assessed clinical, sociodemographic, and territorial factors associated with non-favorable outcomes and mortality.

## 2. Materials and Methods

### 2.1. Study Design and Population

**Figure 1:**
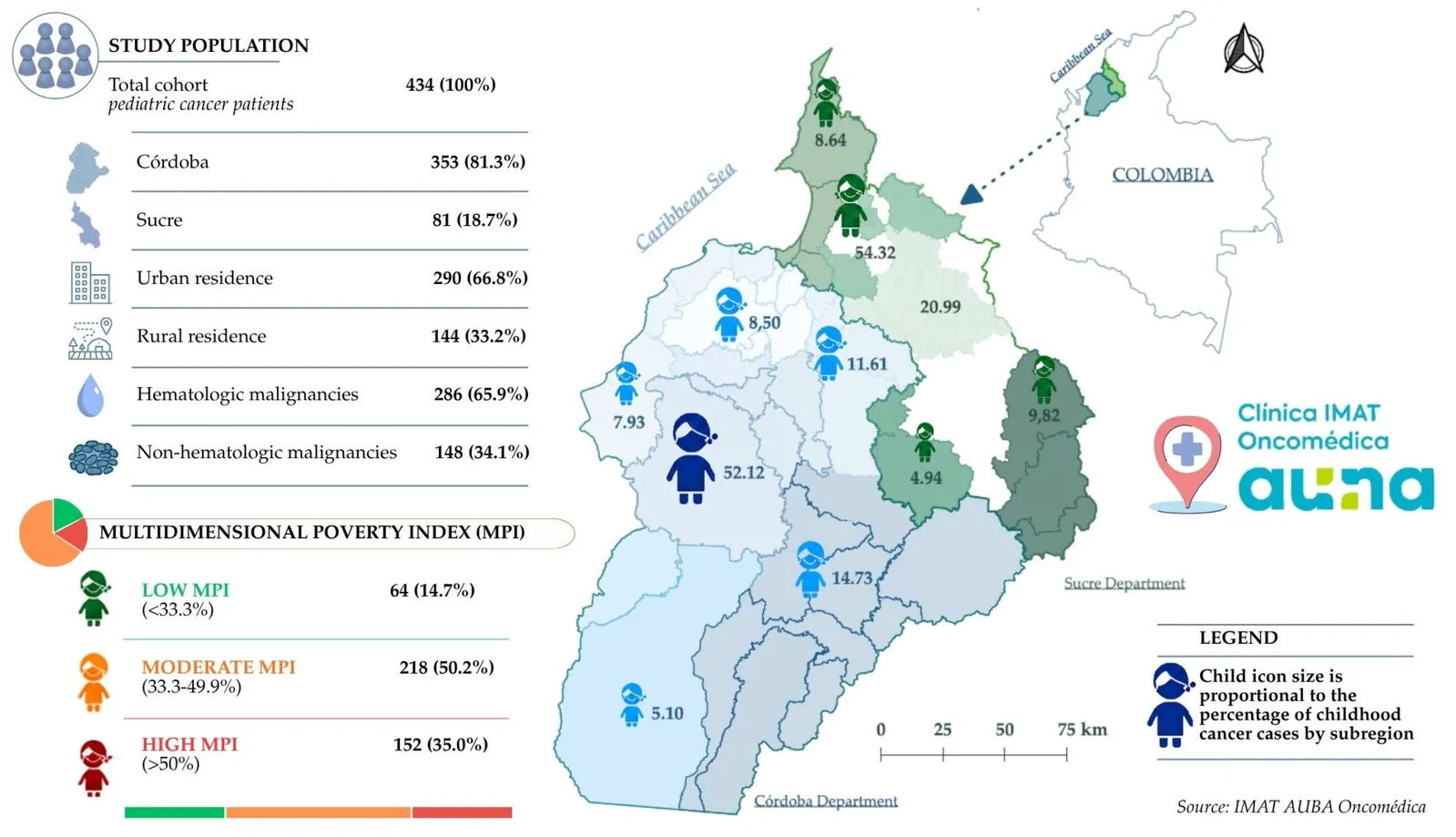
Spatial distribution of pediatric cancer cases across the departments of Córdoba (blue) and Sucre (green), Colombia. The size of each icon is proportional to the percentage of cases originating from each subregion. The map also summarizes the overall distribution of multidimensional poverty index (MPI) categories and key demographic and clinical characteristics of the study cohort (*n* = 434). The referral center providing specialized pediatric oncology care is indicated on the map.

A retrospective hospital-based study was conducted in a pediatric cohort treated between 2018 and 2024 at the Instituto Médico de Alta Tecnología IMAT Oncomédica Auna, located in Montería, Córdoba, Colombia (Figure 1). This institution serves as an oncology referral center for Córdoba and receives patients from neighboring departments, including Sucre, Antioquia, and Bolívar.

The cohort was assembled using the institutional clinical care database and supplemented by an individual review of medical records. Patients’ clinical trajectories were reconstructed using chemotherapy and treatment records, outpatient visits, and hospitalization records.

To define the final study cohort, pediatric patients from birth to 18 years of age who were treated during the study period, had an oncologic diagnosis registered at the institution, and were residents of Córdoba or Sucre were included. In this process, duplicate records, medical records with insufficient information to establish territorial origin or clinical outcome and referred cases without continuity of care or treatment at the institution were excluded. This selection ensured territorial comparability, analytical consistency, and sufficient statistical robustness for subsequent analyses.

Patients were classified by territorial origin at the department, municipality, address, and area of residence levels (urban or rural). This disaggregation enabled a more precise characterization of the cohort’s spatial distribution and the incorporation of geographic and socioeconomic context into subsequent analyses.

Sociodemographic and clinical variables were obtained from the institutional database and from individual medical record reviews. Oncologic diagnoses were classified according to the typology used by the Instituto Nacional de Salud (INS) of Colombia for the surveillance event “Cancer in children under 18 years of age” (INS code 115) [21], which is based on the 12 main diagnostic groups of the International Classification of Childhood Cancer, third edition (ICCC-3) [22]. This classification includes categories such as acute lymphoblastic leukemia, acute myeloid leukemia, other leukemias, lymphomas, and reticuloendothelial neoplasms, central nervous system tumors, malignant bone tumors, soft tissue and extraosseous sarcomas, germ cell tumors, and malignant epithelial tumors, among others. For analytical purposes, these categories were grouped into two broad groups: hematologic malignancies and non-hematologic malignancies.

Clinical outcomes were defined through individual review of each patient’s care trajectory and classified into three categories: favorable, corresponding to patients in complete remission, under oncologic follow-up, or receiving treatment with curative intent; non-favorable, corresponding to patients receiving palliative care or who abandoned treatment; and death. This classification allowed differentiation of patients’ clinical evolution and the therapeutic course of the disease during the observation period.

### 2.2. Social Vulnerability Assessment

Municipal-level Multidimensional Poverty Index (MPI) values were obtained from the national ANDA data repository, derived from the 2018 National Population and Housing Census (CNPV 2018), based on official estimates produced by the National Administrative Department of Statistics (DANE), Colombia [23]. These values were assigned to each patient based on their municipality of residence and further disaggregated by area of residence (urban vs. rural), allowing the incorporation of a contextual, territory-level proxy for socioeconomic vulnerability. Therefore, MPI was interpreted as a measure of territorial deprivation and not as an individual- or household-level indicator of poverty.

The MPI in Colombia is grounded in the Alkire and Foster methodology, which provides a direct measure of poverty based on multiple deprivations, thereby overcoming the limitations of solely income-based approaches [24]. This index integrates five structural dimensions of well-being: (i) educational conditions of the household, (ii) childhood and youth conditions, (iii) health, (iv) employment, and (v) access to public utility services and housing conditions, operationalized through 15 weighted indicators. According to this methodology, a household is classified as multidimensionally poor if it experiences deprivation in at least 33.3% of the indicators, taking into account the assigned weights. This approach makes it possible not only to identify the proportion of households affected by multidimensional poverty, but also to capture the intensity of the deprivations they experience, integrating both dimensions into a composite measure.

For descriptive analyses, MPI values were categorized according to the cutoff points established by the Alkire–Foster methodology and adopted by the United Nations Development Programme (UNDP): low MPI (<33.3%, not multidimensionally poor), moderate MPI (33.3–49.9%, multidimensionally poor), and high MPI (≥50%, severe multidimensional poverty) [25]. These categories reflect increasing levels of accumulated deprivation across the dimensions of education, health, housing, public services, and employment.

In this study, the MPI was used as an indicator of socioeconomic vulnerability, representing the structural determinants of health at the territorial level. Additionally, the indicators within each dimension were disaggregated to characterize vulnerability profiles within the study population more precisely and to examine their relationships with clinical outcomes.

### 2.3. Statistical Analysis

Descriptive analyses were performed to summarize the sociodemographic, clinical, and territorial characteristics of the study population. Quantitative variables were described using means and standard deviations or medians and interquartile ranges, according to their distribution. Categorical variables were summarized as absolute and relative frequencies.

Clinical outcomes were determined through individual review of each patient’s clinical trajectory up to the last available follow-up and were classified into three mutually exclusive categories: favorable outcome, non-favorable outcome, and death. A favorable outcome was defined as complete remission, ongoing oncologic follow-up, or active treatment with curative intent. A non-favorable outcome was defined as transition to palliative care or documented treatment abandonment. Death was considered a separate outcome category. For regression analyses, favorable outcome was used as the reference category, and two independent binary comparisons were performed: non-favorable outcome versus favorable outcome, and death versus favorable outcome.

Variables with a *p*-value ≤ 0.20 in the univariate analysis were considered candidates for the multivariable models. In addition, variables with clinical, epidemiological, or theoretical relevance were retained regardless of their statistical significance in the univariate analysis, in order to avoid excluding potential confounders at an early stage of modeling. Before fitting the multivariable models, multicollinearity among predictors was assessed using the generalized variance inflation factor (GVIF). For variables with more than one degree of freedom, the adjusted measure GVIF^1/(2×D*f*)^ was used. Values below 2 were considered indicative of low multicollinearity. Given the limited number of events in the non-favorable and death outcome categories, Firth’s penalized logistic regression was used instead of conventional maximum-likelihood logistic regression. This method reduces small-sample bias and improves the stability of odds ratio estimates in settings with sparse data or rare outcomes. Adjusted odds ratios (aORs), 95% confidence intervals (95% CIs), and *p*-values were reported for the final models. As a sensitivity analysis, conventional maximum-likelihood logistic regression models were fitted using the same outcome definitions and covariates as the primary Firth-penalized models. The consistency of the MPI estimates across both approaches was used to assess the robustness of the findings.

Statistical significance was set at *p* < 0.05. Data processing and statistical analyses were performed using R software (version 4.5.0; R Foundation for Statistical Computing, Vienna, Austria).

### 2.4. Ethical Considerations

This study was conducted in accordance with Resolution 8430 of 1993 of the Colombian Ministry of Health. According to Article 11, it was classified as research without risk because it involved the analysis of secondary data. The protocol was reviewed and approved by the Institutional Ethics Committee of Universidad del Sinú, Colombia (Act No. 003, 18 April 2023), and by the Ethics Committee of Clínica IMAT Oncomédica Auna, Colombia (Act No. 625, 4 November 2025).

## 3. Results

### 3.1. Sociodemographic Characteristics of the Study Population

A total of 434 pediatric patients with cancer diagnosed between 2018 and 2024 were included in the study (Table 1). The mean age at diagnosis was 10.25 ± 5.34 years, with a median age of 11 years (IQR: 5–15). Male patients accounted for 58.8% of the cohort (*n* = 255), while females represented 41.2% (*n* = 179). The largest age group was adolescents aged 15–18 years (30.0%), followed by children aged 10–14 years (25.3%), 5–9 years (23.5%), and 0–4 years (21.2%).

Regarding clinical characteristics, hematologic malignancies predominated, accounting for 65.9% of all cases (*n* = 286), whereas non-hematologic malignancies represented 34.1% (*n* = 148). Within hematologic malignancies, acute lymphoblastic leukemia was the leading diagnosis, comprising 180 cases, equivalent to 41.5% of the total cohort and 62.9% of hematologic malignancies. Lymphomas and reticuloendothelial neoplasms were the second most frequent hematologic group, with 71 cases (16.4% of the cohort; 24.8% of hematologic malignancies), followed by acute myeloid leukemia with 30 cases (6.9% of the cohort; 10.5% of hematologic malignancies). Other leukemia subtypes were uncommon, representing only 1.2% of the total cohort.

Among non-hematologic malignancies, malignant bone tumors were the most frequent group, with 44 cases, representing 10.1% of the total cohort and 29.7% of non-hematologic malignancies. Central nervous system tumors were the second most frequent non-hematologic diagnosis, accounting for 31 cases (7.1% of the cohort; 20.9% of non-hematologic malignancies), followed by renal neoplasms with 22 cases (5.1%; 14.9%), soft tissue and extraskeletal sarcomas with 19 cases (4.4%; 12.8%), and germ cell, trophoblastic, and gonadal tumors with 18 cases (4.1%; 12.2%). Less frequent diagnostic groups included hepatic tumors, malignant epithelial tumors and melanoma, retinoblastoma, other abdominal tumors, and other unspecified malignant neoplasms, each representing a small proportion of the cohort. At the last follow-up, 360 patients (82.9%) had a favorable clinical status, whereas 37 patients (8.5%) had a non-favorable outcome and 37 patients (8.5%) had died.

From a territorial perspective, most patients resided in the department of Córdoba (*n* = 353; 81.3%), while 81 patients (18.7%) came from Sucre. The geographic distribution of cases across the study area is shown in Figure 1. In Córdoba, cases originated from all major subregions of the department, with higher proportions observed in Middle Sinú, Savannas, and Upper Sinú. Additional cases were identified in Lower Sinú, San Jorge, and the Caribbean Coastal area. In Sucre, cases were distributed across several subregions, with higher concentrations in Montes de María, Savannas, and La Mojana, and smaller proportions from San Jorge and the Gulf of Morrosquillo.

Urban residence predominated in the cohort (66.8%), although one-third of patients lived in rural areas (33.2%). Most participants were enrolled in the subsidized health insurance scheme (78.1%), while 21.9% were affiliated with the contributory scheme.

The mean MPI was 40.50 ± 18.92, with a median of 32.70 (IQR: 21.30–53.30). According to the Alkire–Foster classification, 64 patients (14.7%) lived in territories with low multidimensional poverty, 218 (50.2%) in territories with moderate multidimensional poverty, and 152 (35.0%) in territories with high multidimensional poverty. Overall, 85.2% of the cohort lived in territories classified as having moderate or high multidimensional poverty, indicating that most cases came from territories with substantial contextual socioeconomic vulnerability.

### 3.2. Territorial Multidimensional Vulnerability Profile of the Study Population

The territorial vulnerability profile was assessed using the Colombian Multidimensional Poverty Index (MPI), an official DANE measure based on the Alkire–Foster methodology. Higher MPI values indicate greater simultaneous deprivation across education, childhood and youth conditions, health, employment, housing, and public utilities, reflecting greater structural socioeconomic vulnerability (Table 2).

Overall, the mean MPI was 40.55 ± 18.92. However, rural areas showed a substantially higher MPI than urban areas (62.66 ± 12.67 vs. 29.53 ± 9.52), indicating a greater burden of contextual territorial vulnerability in the rural municipalities and areas from which patients originated.

Educational deprivation was more pronounced in rural territories. Illiteracy was higher in rural than in urban areas (32.65 ± 8.19 vs. 13.21 ± 5.34), as was low educational attainment (73.69 ± 9.16 vs. 41.98 ± 7.99). Within the childhood and youth dimension, rural areas also showed higher mean values for school delay, school absenteeism, and child labor.

In the health dimension, barriers to healthcare access were slightly higher in rural areas than in urban areas (5.11 ± 4.75 vs. 3.98 ± 2.34), whereas the uninsured population was higher in urban areas (16.23 ± 4.12 vs. 13.05 ± 2.35). Employment-related deprivation was high across the cohort, particularly informal employment, which was higher in rural areas (93.73 ± 2.20) than in urban areas (87.22 ± 5.71). Long-term unemployment also showed higher values in rural territories (51.88 ± 8.78 vs. 38.37 ± 6.71).

The largest rural–urban gaps were observed in housing conditions and access to public utilities. Lack of access to improved water sources was markedly higher in rural than in urban areas (42.99 ± 20.20 vs. 6.99 ± 9.85), as was inadequate flooring material (60.11 ± 16.78 vs. 12.21 ± 6.79). Rural areas also showed higher levels of inadequate excreta disposal and inadequate exterior wall material. Severe overcrowding was similar between areas, with slightly higher values in urban territories.

Overall, these findings indicate that the rural territories of origin of pediatric cancer patients showed a substantially higher burden of multidimensional poverty, particularly in domains related to education, employment, housing quality, water access, and sanitation.

### 3.3. Factors Associated with Clinical Outcomes

Factors associated with clinical outcomes were initially explored using univariate logistic regression models. Two independent comparisons were evaluated, using favorable clinical outcome as the reference category: non-favorable clinical outcome versus favorable clinical outcome, and death versus favorable clinical outcome. The variables included at this stage were sex, age at diagnosis, Multidimensional Poverty Index, health insurance scheme, tumor classification, and area of residence. Crude ORs, 95% confidence intervals, and *p*-values are presented in Table 3.

In the comparison between non-favorable clinical outcome and favorable clinical outcome, age at diagnosis showed a statistically significant association. Each additional year of age was associated with an approximately 7% increase in the odds of presenting a non-favorable clinical outcome (OR = 1.07; 95% CI: 1.02–1.13; *p* = 0.003). This finding suggests that, in the individual analysis, patients who were older at diagnosis tended to show a higher relative frequency of non-favorable clinical outcomes compared with those with favorable evolution.

Tumor classification was also significantly associated with clinical outcome. Compared with patients with hematologic malignancies, those with non-hematologic malignancies had higher odds of presenting a non-favorable outcome (OR = 1.84; 95% CI: 1.09–3.11; *p* = 0.023). This result indicates that, before adjustment for other variables, tumor type showed an important relationship with clinical evolution. In contrast, sex, MPI, health insurance scheme, and area of residence were not statistically associated with non-favorable clinical outcome in the univariate analysis.

In the comparison between death and favorable clinical outcome, health insurance scheme and tumor classification were significantly associated with mortality. Patients enrolled in the subsidized health insurance scheme had higher odds of death compared with those enrolled in the contributory scheme (OR = 3.42; 95% CI: 1.03–11.39; *p* = 0.045). Although the confidence interval was wide, this result suggests a possible relationship between health insurance conditions and mortality, which should be interpreted with caution and further evaluated in the multivariable model.

Similarly, patients with non-hematologic malignancies had higher odds of death compared with those with hematologic malignancies (OR = 2.37; 95% CI: 1.16–4.83; *p* = 0.017). This finding reinforces the importance of tumor classification as a clinically relevant variable in explaining outcomes, particularly mortality. Age at diagnosis showed a borderline association with death, with an approximately 7% increase in the odds for each additional year of age; however, this association did not reach conventional statistical significance (OR = 1.07; 95% CI: 1.00–1.14; *p* = 0.053). Sex, MPI, and area of residence were not significantly associated with mortality in the univariate analysis.

Overall, the univariate models showed that age at diagnosis and tumor classification were relevant variables for non-favorable clinical outcome, whereas health insurance scheme, tumor classification, and, to a lesser extent, age at diagnosis showed associations with death. These variables, together with those of clinical, epidemiological, or theoretical relevance, were considered for the construction of the multivariable Firth’s penalized logistic regression models.

Before fitting the multivariable models, multicollinearity among predictors was assessed using the adjusted generalized variance inflation factor. No relevant multicollinearity was identified. Adjusted GVIF values ranged from 1.005 to 1.776 in the non-favorable versus favorable outcome model and from 1.004 to 1.697 in the death versus favorable outcome model.

**Figure 2:**
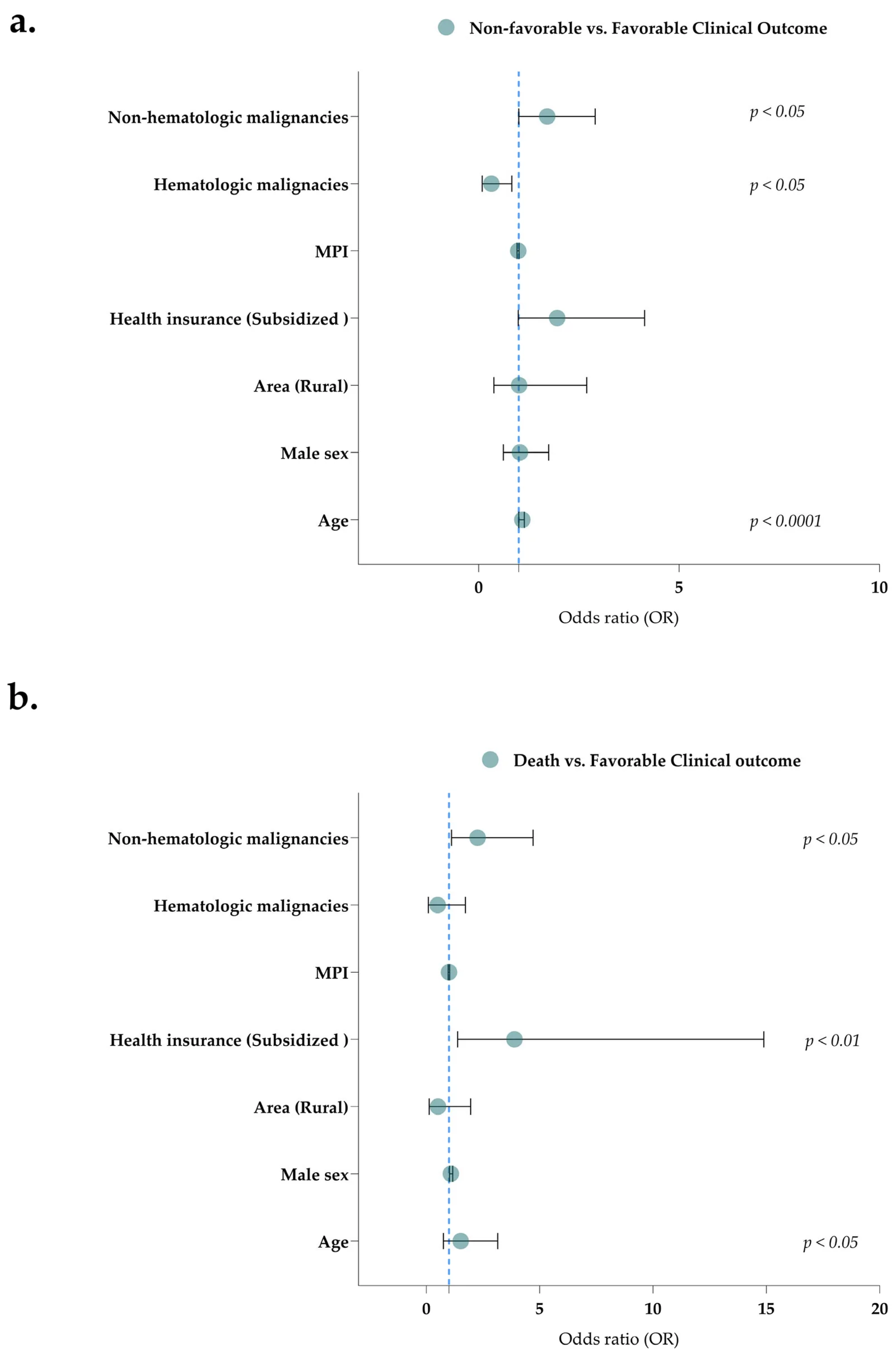
Multivariable Firth’s penalized logistic regression models for clinical outcomes in children with cancer. Adjusted odds ratios (aORs) and 95% confidence intervals (95% CIs) from multivariable Firth’s penalized logistic regression models evaluating factors associated with (**a**) non-favorable clinical outcome versus favorable clinical outcome and (**b**) death versus favorable clinical outcome. Models included sex, age at diagnosis, Multidimensional Poverty Index (MPI), health insurance scheme, tumor classification, and area of residence. The vertical dashed line indicates the null value (aOR = 1). Statistically significant associations (*p* < 0.05) are highlighted.

In the multivariable Firth-penalized logistic regression models (Figure 2), adjusted associations between explanatory variables and clinical outcomes were evaluated considering two independent comparisons: non-favorable clinical outcome versus favorable clinical outcome, and death versus favorable clinical outcome. These models were used to obtain more stable estimates given the limited number of events in the non-favorable and death outcome categories.

In the multivariable Firth-penalized logistic regression model for non-favorable clinical outcome versus favorable outcome, age at diagnosis remained significantly associated with the outcome. Each additional year of age was associated with higher adjusted odds of a non-favorable outcome (aOR = 1.09; 95% CI: 1.03–1.14; *p* = 0.0001). Tumor classification also remained significant: patients with non-hematologic malignancies had higher adjusted odds of non-favorable outcome compared with those with hematologic malignancies (aOR = 1.71; 95% CI: 1.00–2.91; *p* = 0.048). The subsidized health insurance scheme showed a borderline association with non-favorable outcome (aOR = 1.96; 95% CI: 0.99–4.14; *p* = 0.052). Sex, MPI, and rural residence were not independently associated with non-favorable outcome.

In the comparison between death and favorable clinical outcome, age at diagnosis, health insurance scheme, and tumor classification were independently associated with mortality. Each additional year of age was associated with an approximately 8% increase in the adjusted odds of death (aOR = 1.08; 95% CI: 1.014–1.163; *p* = 0.017). Patients enrolled in the subsidized health insurance scheme had higher adjusted odds of death than those in the contributory scheme (aOR = 3.89; 95% CI: 1.39–14.89; *p* = 0.008). Similarly, patients with non-hematologic malignancies had higher adjusted odds of death compared with those with hematologic malignancies (aOR = 2.26; 95% CI: 1.12–4.72; *p* = 0.024). MPI and rural residence were not independently associated with mortality after adjustment.

Overall, Firth’s penalized models suggest that age at diagnosis and tumor classification were relevant factors for both non-favorable clinical outcome and death, whereas subsidized health insurance showed a particularly important association with mortality. Although MPI and area of residence describe a substantial burden of territorial vulnerability in the cohort, these variables did not show an independent association with clinical outcomes after multivariable adjustment.

## 4. Discussion

This study examines regional evidence on the territorial distribution, multidimensional socioeconomic vulnerability, and clinical outcomes of childhood cancer in Córdoba and Sucre, two departments of the Colombian Caribbean characterized by substantial social and territorial inequalities. Overall, these findings represent a relevant contribution to understanding disparities in pediatric oncology in resource-limited settings. Previous studies in low- and middle-income countries have shown that barriers related to timely diagnosis, early referral, access to specialized services, and treatment continuity remain critical challenges for childhood cancer care [6,15,26]. In this context, generating local evidence is essential to guide surveillance strategies, early referral, and comprehensive pediatric oncology care.

One of the main contributions of this study is that it represents one of the first hospital-based reports aimed at characterizing childhood cancer in Córdoba and Sucre from a clinical, territorial, and socioeconomic perspective. The cohort included 434 pediatric patients diagnosed between 2018 and 2024 and treated at a regional referral center, which allowed us to describe the epidemiological and clinical profile of the population, its territorial distribution, and its exposure to multidimensional poverty. Together, these results provide a framework for interpreting the clinical, territorial, and socioeconomic patterns described in the following sections.

Regarding the epidemiological and clinical profile of the cohort, the results showed a predominance of male patients, a mean age at diagnosis of 10.25 years, and a higher proportion of cases among adolescents aged 15–18 years. This age pattern supports the inclusion of adolescents in pediatric cancer surveillance and care strategies, as this group may have clinical, psychosocial, and access-related needs that differ from those of younger children.

Hematologic malignancies were the predominant tumor group, and acute lymphoblastic leukemia was the most frequent diagnosis. This finding is consistent with international evidence recognizing leukemias as the main diagnostic group in childhood cancer, particularly among children younger than 15 years [27]. In Colombia, studies based on population-based cancer registries have highlighted the relevance of childhood leukemia as a priority event for epidemiological surveillance, given its frequency and the variability of incidence trends across regions and periods [28].

Although non-hematologic malignancies represented a smaller proportion of the cohort, they included bone tumors, central nervous system tumors, renal neoplasms, soft tissue sarcomas, and germ cell tumors, among others. This diagnostic heterogeneity may translate into more complex diagnostic and therapeutic pathways, as well as differential needs for referral, imaging, oncologic surgery, radiotherapy, and multidisciplinary management. National studies have also emphasized the importance of analyzing non-leukemia childhood cancers from a territorial perspective, given possible spatial clustering patterns and differences in access to specialized services [13]. The availability of local information on age, sex, tumor type, and diagnostic distribution is therefore necessary to strengthen service planning, guide referral pathways, and complement national systems for monitoring clinical outcomes in childhood cancer [28].

The territorial distribution of cases showed that most patients came from Córdoba, with an important concentration in the Middle Sinú subregion, where Montería and the referral center IMAT AUNA Oncomédica are located. In Sucre, cases were mainly concentrated in Montes de María, Sabanas, and La Mojana. This pattern may be related not only to the population distribution of the departments, but also to the regional organization of health services, referral pathways, and geographic proximity to specialized centers.

The location of IMAT AUNA Oncomédica in Montería may favor the capture of patients from nearby municipalities or from subregions with better connectivity to the departmental capital. International evidence has shown that distance to treatment centers, travel time, and rurality may influence timely access to pediatric oncology care, treatment experiences, continuity of care, and clinical outcomes [6,29,30]. In the United States, distances greater than 50 miles, approximately 80 km, have been associated with lower survival among children and young adults with acute lymphoblastic leukemia [31]. Similarly, recent evidence has shown that children living farther from pediatric cancer centers may face additional access barriers related to travel burden, availability of specialized services, and social determinants of health [32].

This evidence helps contextualize the hospital-based distribution observed in Córdoba and Sucre. The concentration of cases in subregions closer to or better connected with Montería may reflect patterns of accessibility and use of specialized services. Therefore, these findings should be interpreted as a referral-center approximation of the regional burden of childhood cancer rather than as a population-based incidence estimate. In low- and middle-income countries, a systematic review identified travel distance as a determinant of delayed childhood cancer care. Other relevant factors included household income, lack of transportation, rural residence, parental education, and the use of traditional medicine. In these settings, geographic barriers may interact with health-system limitations, treatment-related mortality, and unequal availability of specialized pediatric oncology resources [7,33]. Thus, the observed distribution supports considering geographic and functional accessibility when interpreting service-use patterns in this regional referral setting.

Although the MPI did not show an independent association with clinical outcomes in the adjusted models, the cohort originated largely from territories with substantial structural deprivation. More than 85% of cases came from municipalities classified as having moderate or high multidimensional poverty, and the rural municipalities and areas of origin showed more unfavorable contextual conditions in education, employment, housing, access to improved water sources, and sanitation. These findings suggest that territorial inequalities are part of the structural context in which pediatric oncology care is delivered in the Colombian Caribbean region.

This result is consistent with international evidence recognizing the role of socioeconomic vulnerability in the burden and outcomes of childhood cancer. Recent Global Burden of Disease analyses have shown that the burden of childhood cancer is disproportionately concentrated in countries with lower socioeconomic development, reflecting persistent inequalities in diagnosis, treatment, and access to health services [3]. Likewise, recent studies have indicated that social determinants of health, including educational level, household income, rurality, and access to specialized services, may influence survival and the care trajectory of children with cancer [6,32,34].

Accordingly, the MPI should be interpreted as a contextual marker of territorial vulnerability. It may help identify areas where social and environmental conditions limit effective access to pediatric oncology care, even when no direct association is observed in adjusted models. The present findings may be interpreted as an initial step toward incorporating a One Health perspective into pediatric oncology research in vulnerable territories [35]. Although this study did not evaluate direct environmental exposures or establish environmental causality, the integration of clinical outcomes with municipality-level socioeconomic and contextual indicators highlights the relevance of non-clinical determinants that may influence pediatric cancer care beyond individual tumor-related factors. From this perspective, territorial variables related to poverty, rurality, housing conditions, water access, sanitation, education, employment, and healthcare access barriers may help identify structural conditions that affect the cancer care continuum [19,36]. This approach may support the development of programs and policies aimed at improving pediatric cancer care in referral-based settings, where rurality, infrastructure, transportation, and health-service organization can influence timely diagnosis, treatment continuity, and the ability of families to navigate the healthcare system.

These deprivations have clinical and public health relevance. Globally, inadequate water, sanitation, and hygiene conditions affect approximately 2.4 billion people who use unimproved sanitation facilities and 946 million people who practice open defecation, generating a high burden of gastrointestinal diseases, especially in low- and middle-income countries [37]. In addition, a meta-analysis showed that WASH interventions were associated with a 17% reduction in all-cause childhood mortality and a 45% reduction in mortality from infectious diseases [38]. In sub-Saharan Africa, an analysis of 824,694 children found that improved housing was associated with lower odds of malaria, diarrhea, stunting, underweight, and anemia, with approximate relative reductions ranging from 8% to 18% [39]. These findings are relevant to pediatric oncology, as children undergoing treatment may experience periods of severe immunosuppression during which housing, water, and sanitation conditions may influence the risk of infections and complications.

The absence of a statistically significant association between MPI and clinical outcomes does not imply that territorial conditions are irrelevant. Rather, territorial vulnerability may influence pediatric cancer outcomes indirectly through clinical and health-system pathways not captured in this study. Evidence demonstrates that diagnostic delay negatively affects survival in certain pediatric cancers, particularly acute lymphoblastic leukemia, retinoblastoma, and some solid tumors [40], while treatment abandonment remains a leading cause of treatment failure in low- and middle-income countries, with rates ranging from 24% to 54% and potentially reducing survival by 20–30 percentage points [41]. Other unmeasured pathways include treatment interruption, relapse, distance from the treatment center, financial toxicity, social support, treatment adherence, and quality of life. The absence of these variables may have introduced residual confounding and may partly explain why MPI was not independently associated with outcomes in the adjusted models. Consequently, MPI should be interpreted as a contextual marker of territorial vulnerability and health sustainability, rather than as an isolated clinical predictor [42]. Its incorporation into childhood cancer studies can help identify territories where social and environmental conditions may limit effective access to care, even when their effect is not expressed as a direct association in adjusted clinical models.

To explore factors associated with clinical outcomes, adjusted analyses were performed using penalized logistic regression. The results allowed the identification of variables associated with the probability of non-favorable outcomes and mortality, providing evidence on clinical and sociodemographic characteristics that may influence patient evolution.

In the adjusted models, age at diagnosis and tumor classification emerged as factors related to the probability of presenting a non-favorable outcome. Each additional year of age increased the adjusted odds of a non-favorable outcome, and patients with non-hematologic malignancies had a higher probability of non-favorable evolution compared with those with hematologic malignancies. This finding is consistent with international evidence documenting variability in survival according to age at diagnosis. In acute lymphoblastic leukemia, survival tends to be highest among children aged 1–4 years and progressively declines at older ages, whereas infants younger than one year have poorer outcomes in both leukemias and solid tumors [43,44]. Similarly, European population-based studies have shown that, excluding retinoblastoma, five-year survival is slightly higher among children aged 1–4 years than among infants or older children; in addition, patients aged 10–14 years with lymphoid leukemia or rhabdomyosarcoma have lower survival than younger children with the same diagnosis [45]. In this sense, age may act as a marker of clinical and care-related differences associated with disease presentation, diagnostic timeliness, tumor type, therapeutic complexity, and differential care needs among adolescents and older children.

Age at diagnosis may also reflect developmental vulnerabilities that extend beyond tumor biology. Pediatric cancer affects children at different stages of neurocognitive, emotional, educational, and social development, which may influence symptom recognition, treatment adherence, supportive care needs, and long-term survivorship [46]. Younger children may be more vulnerable to treatment-related neurocognitive effects, particularly in attention, executive function, processing speed, and learning [47], whereas adolescents and young adults may face additional challenges related to autonomy, emotional distress, body image, fertility concerns, school disruption, social isolation, and transition between pediatric and adult-oriented care [48,49]. These developmental factors may partly explain why older age was associated with poorer outcomes in this cohort, together with tumor biology, diagnostic complexity, and health-system barriers [49].

The association between non-hematologic malignancies and non-favorable outcomes should be interpreted considering the heterogeneity of this diagnostic group. This category includes central nervous system tumors, bone tumors, sarcomas, renal neoplasms, and germ cell tumors, among others. These malignancies may require more complex diagnostic and therapeutic pathways, including oncologic surgery, specialized imaging, radiotherapy, multidisciplinary support, and prolonged follow-up. International population-based evidence shows important differences in survival according to tumor type and region. In the SURVCAN-3 study, which included 16,821 children with cancer diagnosed between 2008 and 2017 in Africa, Asia, Latin America, and the Caribbean, three-year survival for leukemia ranged from 30.4% in Kenya to 89.5% in Puerto Rico, whereas survival for central nervous system tumors ranged from 32.0% in Algeria to 79.3% in Puerto Rico [50]. These differences reflect the interaction between tumor biology, diagnostic capacity, therapeutic availability, and the organization of care services.

Regarding mortality, the penalized models showed significant associations with age at diagnosis, subsidized health insurance scheme, and non-hematologic malignancies. The association with older age may reflect differences in tumor biology, stage at diagnosis, delayed clinical suspicion, or specific access barriers among adolescents, a group that may face complex transitions between pediatric and adult care pathways [45]. In turn, the higher mortality observed among patients with non-hematologic malignancies reinforces the need to consider the diagnostic and therapeutic complexity of these tumors, as well as the timely availability of specialized services [50].

The subsidized health insurance scheme showed a significant association with mortality (aOR = 3.89; 95% CI: 1.39–14.89); however, this result should be interpreted with caution. The wide confidence interval indicates limited precision in the effect estimate, likely related to the small number of deaths and the unequal distribution of patients between insurance groups. Insurance scheme should not be understood as a direct cause of death, but rather as a possible marker of social vulnerability, unfavorable economic conditions, and differential access barriers within the health system. In Colombia, clinical surveillance evidence in childhood cancer has highlighted the importance of analyzing outcomes under real-world care conditions and considering differences related to insurance scheme, diagnostic timeliness, and treatment continuity [14].

This study has relevant implications for Córdoba and Sucre by identifying subregions where hospital-based care for children with cancer is concentrated and where surveillance, early diagnosis, and timely referral actions could be prioritized. Integrating territorial distribution with the MPI may help identify areas where families face cumulative barriers related to rurality, transportation, socioeconomic conditions, and access to specialized services. In this regard, the results support the need to strengthen regional pathways for clinical suspicion, referral and counter-referral mechanisms, transportation support, family accompaniment, and strategies that promote treatment continuity.

In addition, this study contributes to the emerging use of multidimensional poverty metrics in clinical research in Latin America. Although the MPI is widely used for policy purposes, its application in pediatric oncology remains limited. In this sense, the findings suggest that multidimensional measures may capture structural patterns of vulnerability that are not always reflected by traditional indicators. However, their interpretation requires caution given their ecological nature.

Among the main limitations, it should be noted that, as a hospital-based study, the results reflect the population captured and treated by the referral center and should therefore not be interpreted as population-based incidence estimates for Córdoba and Sucre. In addition, the MPI was assigned at the municipal and area-of-residence level, rather than at the individual or household level, which may result in unmeasured heterogeneity within territories. Important clinical and care-related variables were also unavailable, including stage at diagnosis, diagnostic and treatment delays, treatment abandonment or interruption, relapse, actual distance from the treatment center, adherence, family support, financial toxicity, infectious complications, and quality of life. Their absence may have introduced residual confounding and may partly explain why MPI was not independently associated with outcomes in the adjusted models, particularly if territorial vulnerability affects outcomes indirectly through delayed diagnosis, treatment discontinuity, or other health-system barriers.

Finally, this work strengthens the evidence base on childhood cancer in socially and environmentally vulnerable territories of the Colombian Caribbean. The results show that integrating clinical data, hospital-based outcomes, and territorial indicators may contribute to a broader understanding of inequalities in pediatric oncology. This approach can inform surveillance processes, territorial prioritization, and equity-oriented interventions, particularly in regions where specialized care depends on referral centers and where socioeconomic conditions may influence the full care trajectory of children with cancer.

## 5. Conclusions

This study provides one of the first hospital-based assessments of childhood cancer in Córdoba and Sucre that integrates clinical outcomes with territorial socioeconomic vulnerability. The cohort exhibited a high burden of multidimensional poverty, with 85.2% of patients residing in areas classified as moderate or high MPI, and marked rural–urban inequalities across education, employment, housing, water access, and sanitation.

Older age at diagnosis and non-hematologic malignancies were consistently associated with poorer clinical outcomes, while subsidized health insurance was independently associated with mortality. Although the MPI did not show an independent association with outcomes after adjustment, its descriptive value remains substantial: it reveals the structural context in which pediatric cancer care occurs and identifies territories where barriers to timely diagnosis and treatment initiation may be concentrated.

These findings may inform clinical practice and health policy by supporting territorial surveillance, earlier referral pathways, transportation and family-support strategies, and equity-oriented pediatric oncology interventions in regions dependent on referral centers. From a One Health perspective, the results also highlight the need to consider social, environmental, and health-system conditions as interconnected determinants of pediatric cancer care.

These results should be interpreted considering the retrospective hospital-based design, the single-center setting, the ecological assignment of MPI at the municipality and area-of-residence level, and the potential for selection bias. Future multicenter and population-based studies should evaluate diagnostic delays, treatment initiation times, distance to care, treatment abandonment, relapse, infectious complications, family financial burden, and quality of life to better understand how territorial vulnerability affects the full pediatric cancer care trajectory.

## Figures and Tables

**Table 1:** Sociodemographic, clinical, and territorial characteristics of children with cancer treated at a referral center in northern Colombia.

Variable	*n* (%) or Mean ± SD
**Sociodemographic characteristics**	
Sex	
Female	179 (41.2)
Male	255 (58.8)
Age at diagnosis (years) Mean ± SD	10.25 ± 5.34
Age at diagnosis (years) Median (IQR)	11 (5–15)
Age group	
0–4 years	92 (21.2)
5–9 years	102 (23.5)
10–14 years	110 (25.3)
15–18 years	130 (30.0)
**Clinical characteristics**	
Tumor classification	
Hematologic malignancies	286 (65.9)
Non-hematologic malignancies	148 (34.1)
Diagnostic group	
Acute lymphoblastic leukemia	180 (41.5)
Acute myeloid leukemia	30 (6.9)
Central nervous system tumors4969824	31 (7.1)
Germ cell, trophoblastic and gonadal tumors	18 (4.1)
Hepatic tumors	5 (1.2)
Lymphomas and reticuloendothelial neoplasms	71 (16.4)
Malignant bone tumors	44 (10.1)
Malignant epithelial tumors and melanoma	4 (0.9)
Other abdominal tumors	1 (0.2)
Other leukemias	5 (1.2)
Other malignant epithelial tumors and melanomas	2 (0.5)
Other neoplasms and unspecified malignant neoplasms	1 (0.2)
Renal neoplasms	22 (5.1)
Retinoblastoma	1 (0.2)
Clinical status at last follow-up	
Favorable	360 (82.9)
Non-favorable	37 (8.5)
Death	37 (8.5)
**Territorial and socioeconomic characteristics**	
Department of residence	
Córdoba	353 (81.3)
Sucre	81 (18.7)
Area of residence	
Urban	290 (66.8)
Rural	144 (33.2)
**Health insurance scheme**	
Contributory	95 (21.9)
Subsidized	339 (78.1)
Multidimensional Poverty Index (MPI)	
Mean ± SD	40.50 ± 18.92
Median (IQR)	32.70 (21.30–53.30)
MPI category	
Low MPI (<33.3%)	64 (14.7)
Moderate MPI (33.3–49.9%)	218 (50.2)
High MPI (≥50%)	152 (35.0)

**Table 2:** Distribution of socioeconomic vulnerability indicators across rural and urban settings in pediatric cancer patients from Córdoba and Sucre.

Indicator	Overall (Mean ± SD)	Urban	Rural
		(Mean ± SD)	(Mean ± SD)
MPI *			
MPI (%)	40.55 ± 18.92	29.53 ± 9.52	62.66 ± 12.67
Educational conditions			
Illiteracy	19.66 ± 11.18	13.21 ± 5.34	32.65 ± 8.19
Low educational attainment	52.50 ± 17.14	41.98 ± 7.99	73.69 ± 9.16
**Childhood and youth conditions**			
Barriers to early childhood services	2.19 ± 2.88	1.96 ± 3.41	2.65 ± 1.17
School delay	18.71 ± 5.39	16.50 ± 3.12	23.14 ± 6.22
School absenteeism	4.38 ± 3.37	3.66 ± 3.52	5.82 ± 2.48
Child labor	1.25 ± 4.31	0.84 ± 0.80	2.07 ± 7.35
**Health conditions**			
Uninsured population	15.18 ± 3.92	16.23 ± 4.12	13.05 ± 2.35
Barriers to healthcare access	4.35 ± 3.38	3.98 ± 2.34	5.11 ± 4.75
**Employment conditions**			
Informal employment	89.38 ± 5.72	87.22 ± 5.71	93.73 ± 2.20
Long-term unemployment	42.85 ± 9.80	38.37 ± 6.71	51.88 ± 8.78
**Housing conditions and public utilities**			
Severe overcrowding	17.19 ± 4.46	17.62 ± 4.28	16.32 ± 4.71
No access to improved water source	18.93 ± 22.08	6.99 ± 9.85	42.99 ± 20.20
Inadequate excreta disposal	29.03 ± 20.87	24.46 ± 19.16	38.24 ± 21.19
Inadequate exterior wall material	10.21 ± 12.35	7.41 ± 5.23	15.84 ± 18.94
Inadequate flooring material	28.10 ± 25.17	12.21 ± 6.79	60.11 ± 16.78

* MPI indicators correspond to territory-level estimates assigned by municipality and area of residence. Values describe the socioeconomic context of the territories of origin and should not be interpreted as individual-level measurements.

**Table 3:** Univariate logistic regression models for factors associated with clinical outcomes.

Non-Favorable vs. Favorable Clinical Outcome	β Coefficient	OR	CI	*p*-Value
Sex _Male vs. female (ref.)_	−0.098	0.91	(0.55–1.51)	0.701
MPI	−0.007	0.99	(0.98–1.01)	0.21
Age	0.072	1.07	(1.02–1.13)	**0.003**
Health insurance scheme _Subsidized vs. contributory (ref.)_	0.555	1.74	(0.88–3.45)	0.112
Hematologic malignancies (ref.)	−1.11	0.33	(0.11–0.97)	0.042
Non-hematologic malignancies	0.608	1.84	(1.09–3.11)	**0.023**
Area of residence _Rural vs. urban (ref.)_	−0.192	0.83	(0.48–1.43)	0.48
**Death vs. Favorable Clinical Outcome**				
Sex _Male vs. female (ref.)_	0.282	1.33	(0.66–2.70)	0.431
MPI	−0.013	0.99	(0.97–1.01)	0.183
Age	0.065	1.07	(1.00–1.14)	0.053
Health insurance scheme _Subsidized vs. contributory (ref.)_	1.229	3.42	(1.03–11.39)	**0.045**
Hematologic malignancies (ref.)	−0.876	0.42	(0.09–1.89)	0.254
Non-hematologic malignancies	0.864	2.37	(1.16–4.83)	**0.017**
Area of residence _Rural vs. urban (ref.)_	−0.636	0.53	(0.24–1.19)	0.124

OR: odds ratio; CI: confidence interval. Favorable clinical outcome was the reference outcome in both comparisons. Reference categories for categorical predictors were female sex, contributory health insurance scheme, hematologic malignancies, and urban area of residence. Age and MPI were modeled as continuous variables; their ORs represent the change associated with a one-year and one-percentage-point increase, respectively. Statistically significant results are shown in bold (*p* < 0.05), whereas borderline associations are underlined (0.05 ≤ *p* < 0.10).

## Data Availability

The raw data supporting the conclusions of this article will be made available by the authors on request.
